# Flexible Planar Monopole Built-in GIS PD Sensor Based on Meandering Technology

**DOI:** 10.3390/s22114134

**Published:** 2022-05-29

**Authors:** Shuo Zhang, Guozhi Zhang, Changyue Lu, Hanlv Tian, Jianben Liu, Xiaoxing Zhang

**Affiliations:** 1 Hubei Engineering Research Center for Safety Monitoring of New Energy and Power Grid Equipment, Hubei University of Technology, Wuhan 430068, China; 1910201114@hbut.edu.cn (S.Z.); 102100219@hbut.edu.cn (C.L.); 102110326@hbut.edu.cn (H.T.); xiaoxing.zhang@outlook.com (X.Z.); 2 State Key Laboratory of Power Grid Environmental Protection, China Electric Power Research Institute, Wuhan 430074, China; liujianben@epri.sgcc.com.cn

**Keywords:** gas-insulated switchgear, partial discharge, flexible antenna, meandering technology

## Abstract

To address the problem of low space utilization of existing rigid Ultra-High Frequency (UHF) sensors for partial discharge (PD) in Gas-Insulated Switchgears (GIS) and the problem of disrupting the electric field distribution inside the GIS. This paper draws on the idea of flexible wearable antennas and introduces planar monopole antennas commonly used in the communication field as GIS PD detection sensors and carried out research on flexible planar monopole sensing technology built into GIS PD. The VSWR of monopole antenna in the UHF low band is optimized by the meandering technique. The size of the designed flexible antenna is 142 mm × 195 mm × 0.28 mm. The simulation and physical test results show that the improved monopole antenna with meandering technology has a VSWR of ≤2 in the frequency bands 570 MHz–830 MHz, 1.38 GHz–1.8 GHz, and 2.2 GHz–2.76 GHz when the bending radius is 0 mm, 200 mm, and 400 mm, respectively. The VSWR in the frequency band 450 MHz–3 GHz is ≤5. A 220 kV GIS PD detection platform was built to test the performance of the designed antenna, and the results showed that the antenna could detect the PD signal after bending deformation with a high Signal Noise Ratio (SNR).

## 1. Introduction

Gas-Insulated Switchgears (GIS) have been widely used in power systems for their advantages of small footprint, good insulation performance, and convenient installation [1,2,3]. The insulation status of GIS is closely related to the safe and stable operation of the power system. According to the CIGRE 23.10 working group survey, the phenomenon of partial discharge (PD) is an important cause of the deterioration of the insulation performance of GIS. When the PD phenomenon occurs within the GIS, Ultra-High Frequency (UHF) electromagnetic signals (frequency range from 300 MHz to 3 GHz) will be generated. UHF PD detection sensors offer high sensitivity by detecting UHF signals while avoiding interference from corona discharges in the environment (below 200 MHz). Therefore, using the UHF method can provide a basis for the effective evaluation and early warning of GIS operation status [4,5].

The UHF method relies on GIS PD detection antenna sensors to detect PD sources. The currently studied GIS PD detection antenna sensors can be divided into external sensors and built-in sensors [3,6,7]. Built-in sensors are generally placed inside the GIS cavity by adding a flange in the form of a hole in the GIS [8], as the GIS metal cavity has a shielding effect on external electromagnetic interference, making its sensitivity and anti-interference ability significantly higher than external sensors [9,10], which has become the standard equipment for GIS of 220 kV and above voltage levels in operation [11]. A built-in snowflake patch fractal antenna for partial discharge detection of high-voltage switchgear was studied in the literature [10], and the antenna can effectively detect the PD signal after being built into the high-voltage switchgear. Literature [12] designed a built-in miniaturized UHF LS Peano fractal antenna with an antenna size of 27.6 × 27.6 × 2 mm, which was placed in GIS through structural transformation. The antenna can effectively detect PD signals. However, most of the currently studied built-in GIS PD detection antennas are rigid substrates, and the antennas placed inside the GIS cannot be conformal to the GIS curved inner wall, which leads to the low space utilization of the built-in sensors and the risk of disrupting the electric field distribution inside the GIS [13].

In order to address the problems of the current GIS PD detection built-in UHF sensor, this paper draws on the widely used planar monopole antenna in the communication field as the GIS PD detection antenna and combines the meandering technology to improve the structure and optimize the performance of the antenna. This paper adopts the flexible material polyetherimide (PI) as the antenna substrate to carry out research on the flexible planar monopole sensor built-in GIS PD based on the meandering technology. Ansoft HFSS was used to build a 3D model of the antenna, and the parameters of the monopole antenna meandering slotting were optimized by the joint optimization method of multi-dimensional parameters. Finally, the designed antenna was physically fabricated, and the PD simulation experiment platform was built to test the antenna’s PD detection performance.

## 2. Antenna Design

### 2.1. Monopole Antenna Body Design

Monopole antenna has the advantages of small size and simple structure, widely used in the field of communication [14,15]. The monopole antenna is usually made on an insulating dielectric substrate, and the top surface of the substrate is laser engraved to make a metal patch as the radiating surface of the antenna to receive the electromagnetic signals propagating in space. The bottom surface of the substrate is covered with copper as the antenna ground. Monopole antennas are usually fed by coaxial probes, coplanar waveguides, or microstrip lines [16]. The monopole antenna dielectric substrate designed in this paper is a rectangular flexible material polyetherimide (PI) with a dielectric constant ($\varepsilon_{r}$) = 3.5 and dielectric loss (tanδ) = 0.02. The thickness of the dielectric substrate is 0.28 mm. Based on the chosen dielectric substrate parameters, the width and length of the monopole antenna metal patch can be calculated from Equation [17]. Where the width a can be calculated from Equation (1):

(1) $$a=\frac{c}{2f_{m}}{\left(\frac{\varepsilon_{r}+1}{2}\right)}^{-1/2}$$

where c is the speed of light, $\varepsilon_{r}$ is the relative permittivity of the dielectric substrate, and $f_{m}$ is the center frequency of the antenna’s operating band. Since the energy concentration of UHF signals is in the frequency band of 500 MHz to 1.5 GHz, the antenna is designed for 900 MHz, according to the literature [18]. The monopole antenna metal patch width a is calculated to be 105 mm according to Equation (1).

The monopole antenna metal patch length b can be calculated from the Equation (2):

(2) $$b=\frac{c}{2f_{m}\sqrt{\varepsilon_{e}}}$$

where $f_{m}$ is the center frequency of the antenna’s operating band. $\varepsilon_{e}$ is the effective dielectric constant, which can be determined from the following equation:

(3) $$\varepsilon_{e}=\frac{\varepsilon_{r}+1}{2}+\frac{\varepsilon_{r}-1}{2}{\left(1+12\frac{h}{a}\right)}^{-1/2}$$

where $\varepsilon_{r}$ is the relative permittivity of the dielectric substrate, *h* is the thickness of the dielectric substrate parameters, and a is the width of the monopole antenna metal patch. Combining the calculation result and the microstrip line simulation, this paper selects the metal patch length b as 159.8 mm. The monopole antenna designed in this paper is fed by SMA coaxial line. The characteristic impedance of the SMA coaxial line is 50 Ω. In order to solve the impedance matching problem, this paper adopts the combination of the microstrip transmission line and the antenna background to achieve impedance matching, in which the characteristic impedance of the microstrip transmission line needs to be designed as 50 Ω. The microstrip transmission line width w and the microstrip transmission line characteristic impedance z satisfy Equation [19]:

(4) $$z=\frac{120\pi }{\sqrt{\varepsilon_{e}}[\frac{w}{h}+2.42-0.44\frac{h}{w}+{\left(1-\frac{h}{w}\right)}^{6}]}$$

The microstrip transmission line width can be calculated by bringing the characteristic impedance of the microstrip line (50 Ω), the dielectric substrate thickness h, and the effective dielectric constant $\varepsilon_{e}$ into the equation. Combined with the calculation of the equation and the optimization of the microstrip line simulation, the microstrip transmission line width w is designed to be 0.3 mm in this paper.

**Figure 1:**
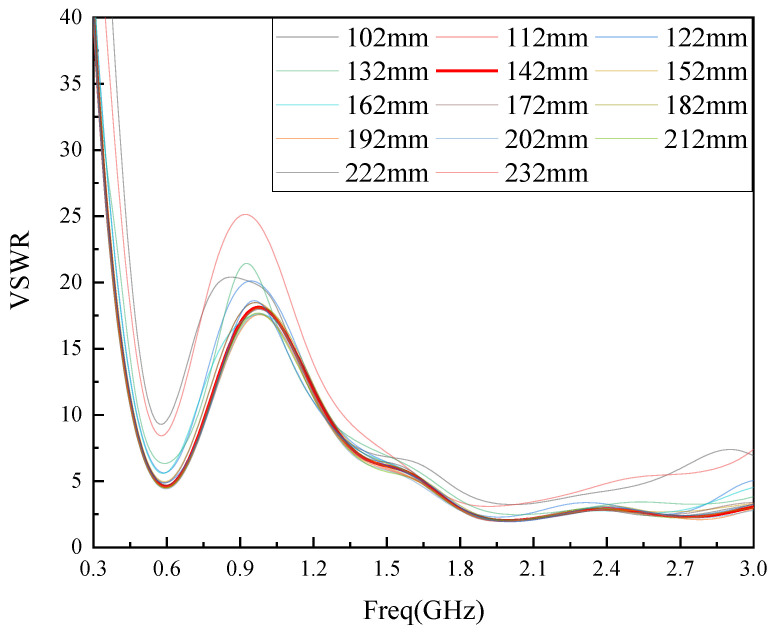
$L_{2}$ parameter simulation optimization diagram.

For selecting the size of the parameters for the length $L_{1}$ and width $L_{2}$ of the flexible dielectric substrate, this paper draws on the model in the literature [19]. In the literature [19], an external rigid base monopole antenna was designed for GIS PD detection. The antenna dielectric substrate is FR-4, the length of the dielectric substrate is 195 mm, the width is 235 mm, and the working frequency of the antenna is 0.45 GHz to 2.8 GHz. In this paper, the length of the media base $L_{1}$ is chosen to be 195 mm, which is consistent with the literature. For the selection of the dielectric base width $L_{2}$, as the antenna designed in this paper is a built-in antenna, the antenna size should meet the miniaturization principle as far as possible, so the dielectric base width parameter $L_{2}$ is reduced based on the literature [19], and the optimal value of $L_{2}$ is found in the range of 102 mm–232 mm every 10 mm for the interval, the simulation results are shown in Figure 1. 

As can be seen from Figure 1, $L_{2}$ size will have an impact on the antenna VSWR performance, when $L_{2}$ is less than 142 mm, the antenna VSWR performance is poor, but when $L_{2}$ is larger than 142 mm, as $L_{2}$ increases, the antenna VSWR performance is close, considering the antenna size, so choose $L_{2}$ = 142 mm as the size of $L_{2}$ parameter in this paper.

**Figure 2:**
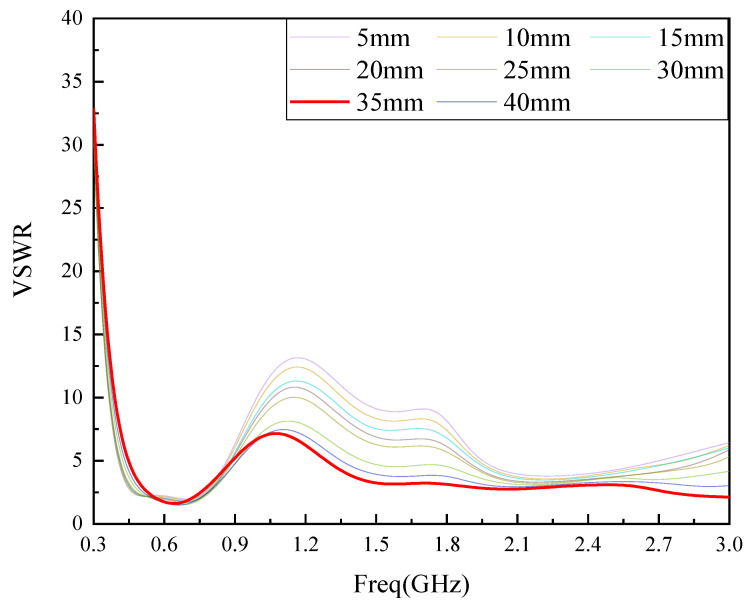
$L_{3}$ parameter simulation optimization diagram.

The monopole antenna electric field is mainly distributed in the antenna metal patch and the antenna background plate. The size of the ground plate involves the antenna metal radiation surface and ground signal-coupling. When the ground plate is too large, it will affect the antenna gain, and when it is too small, it will lead to antenna impedance matching performance decline [20]. In this paper, the length of the grounding plate is chosen to correspond to the width $L_{2}$ of the media substrate, with a size of 142 mm. In order to reduce the coupling area between the ground plate and the radiating metal surface, this paper simulates and optimizes the antenna ground plate width $L_{3}$ in the range of 5 mm to 40 mm, and finds the most available value of $L_{3}$ by VSWR. The optimization results are shown in Figure 2.

**Figure 3:**
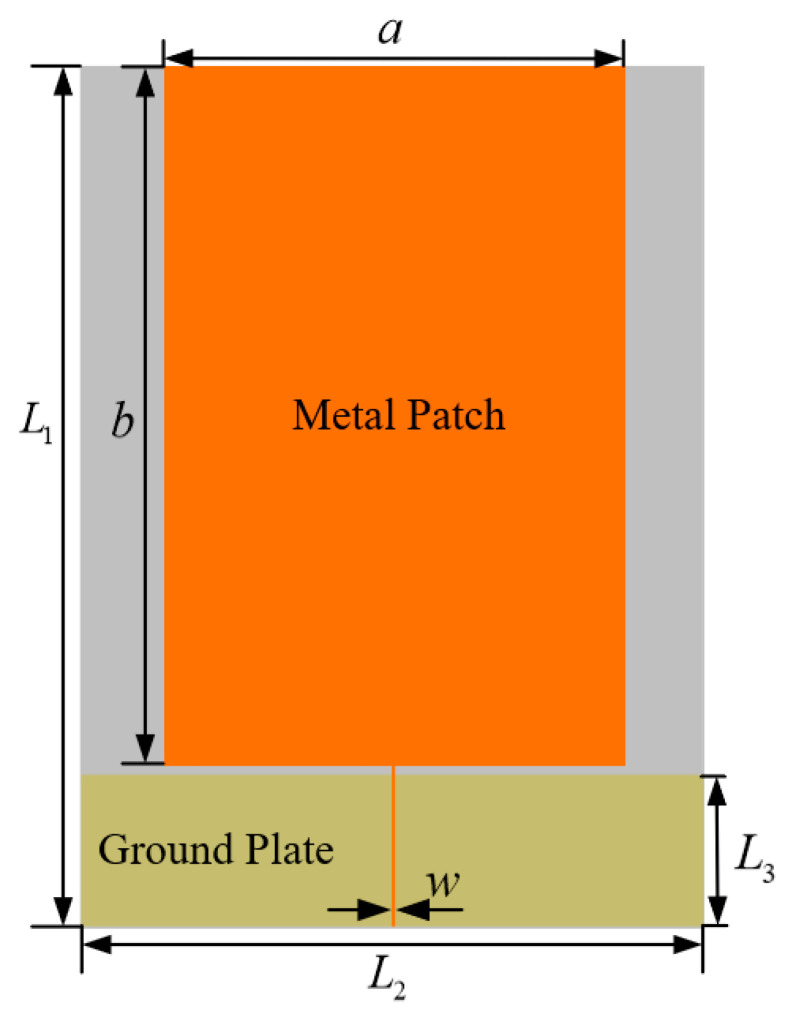
Unimproved monopole antenna.

As can be seen from the figure, with the increase in $L_{3}$, the antenna VSWR gradually decreases, but the degree of reduction gradually decreases, when $L_{3}$ is 35 mm, the antenna VSWR in most of the UHF band VSWR effect is the best, so choose $L_{3}$ size is 35 mm. The overall structure of the unmodified antenna is shown in Figure 3.

### 2.2. Structural Optimization Based on Meandering Technology

Conventional monopole antennas are mostly used in communications and operate at a minimum frequency of 3 GHz and above. If monopole antennas are to be used for GIS PD detection, it is necessary to use broadband technology to expand the working bandwidth of the monopole antenna in the UHF frequency band. In the literature [21], an ultra-wideband antenna with compact size was designed using a time-domain finite-difference algorithm and a genetic algorithm. In literature [22], a gradient feed line was designed to increase the bandwidth of the antenna and improve the impedance matching within the effective bandwidth, resulting in a planar octagonal ultra-wideband antenna with good performance. In this paper, the structure of the monopole antenna designed in Figure 3 is optimized by meandering technology. Meandering technology refers to slotting or etching slits on the metal patch of the microstrip antenna to increase the length of the current flow through the radiating surface of the antenna without increasing the original size of the antenna. When the length of the antenna surface current path increases, it is equivalent to an increase in the effective size of the metal patch, which reduces the minimum cutoff frequency of the antenna operating band and extends the bandwidth of the antenna in the UHF band [17]. 

**Figure 4:**
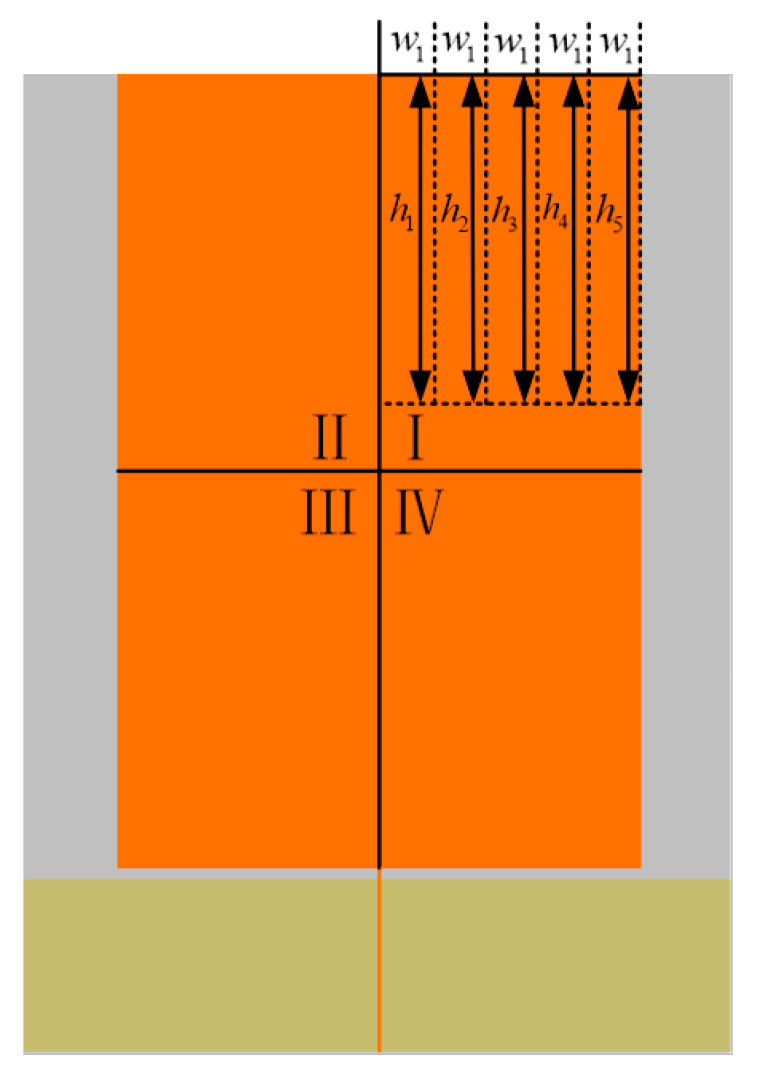
Optimization Diagram of Meandering Technology.

The monopole antenna is usually an axially symmetric structure, and the original axially symmetric structure of the antenna remains unchanged when the meandering technology is used for optimization. Therefore, the same form of meandering technology is applied to the left and right parts of the antenna. Based on the model of the literature [19], the top and bottom sides of the antenna metal radiation patch are treated simultaneously with the meandering technology. Referring to the optimization method of meandering technology in the literature [19], the metal patch is divided into four regions: I, II, III, and IV. Since the antenna model performs the same form of meandering processing in the four regions simultaneously, it is only necessary to optimize the meandering slotting parameters in region I. As shown in Figure 4, the area I is divided into five rectangles of equal width, the width is represented by $w_{1}$ ($w_{1}$ = 10.47 mm can be calculated), and the lengths of the five rectangles are $h_{1}$, $h_{2}$, $h_{3}$, $h_{4}$, $h_{5}$. By optimizing the length of each rectangle to increase the current flow path, the antenna UHF band VSWR performance is optimized. 

**Figure 5:**
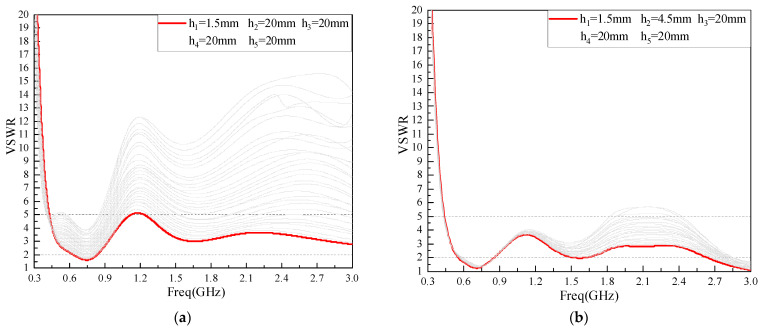

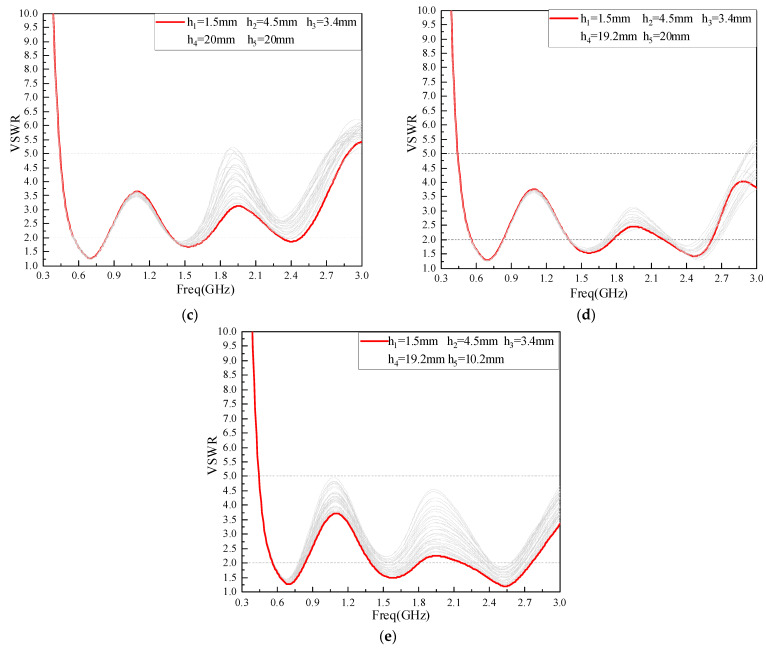
$h_{1}$~$h_{5}$ Simulation diagram for parameter optimization: (**a**) $h_{1}$; **(b**) $h_{2}$; (**c**) $h_{3}$; (**d**) $h_{4}$; (**e**) $h_{5}$.

**Figure 6:**
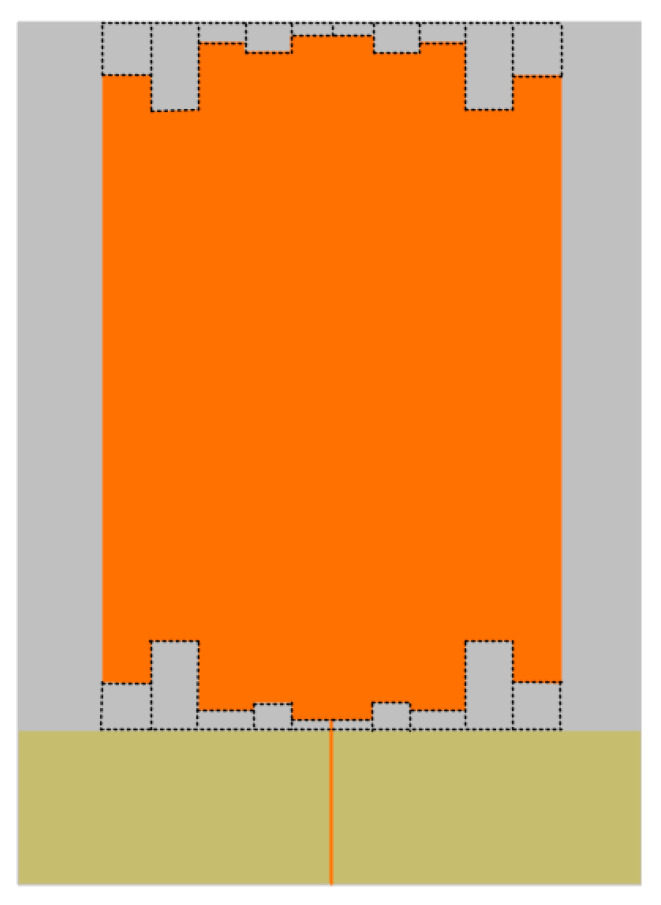
Model diagram of improved monopole antenna.

The antenna model is established by HFSS software, and the five rectangle length parameters $h_{1}$~$h_{5}$ are optimized, respectively. In order to ensure the feasibility of the optimization scheme, the optimization method used in this paper is as follows: With the other four parameters selected as fixed values of H (note: H = 20 mm was selected empirically), a VSWR simulation was performed for the other parameter to be optimized in the range 0 mm–20 mm, and the optimal value of this optimized parameter was obtained by comparing the bandwidth sizes with VSWR less than 2 in the 300 MHz–3 GHz frequency band; On this basis, three of the remaining four parameters to be optimized are selected as fixed values of H. Carry out the simulation analysis of the VSWR in the range of 0 mm–20 mm for the other parameter of the four parameters to be optimized, and obtain the optimal value of the optimized parameter; By analogy, the optimal values of the five rectangular length parameters $h_{1}$~$h_{5}$ are obtained. The results for the five rectangular length parameters from $h_{1}$~$h_{5}$ are shown in Table 1, where the simulation plots for the five rectangular length parameters from $h_{1}$~$h_{5}$ are shown in Figure 5. The improved antenna model of the meandering technology structure is shown in Figure 6.

**Figure 7:**
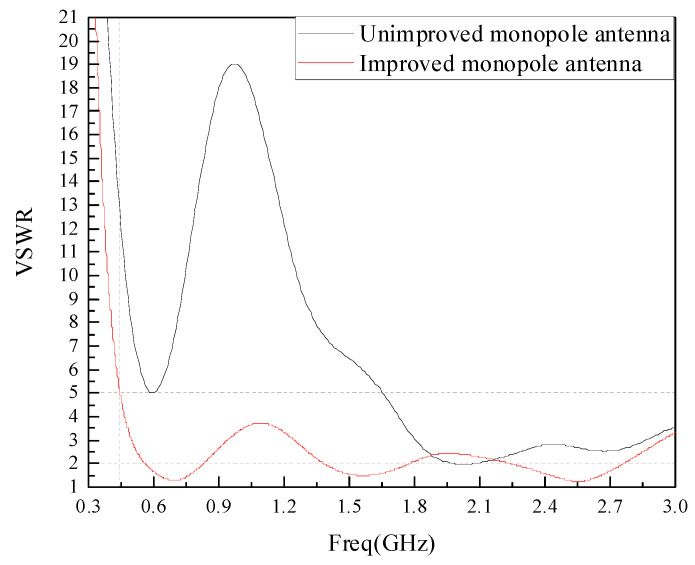
Antenna VSWR before and after improvement.

The VSWR of the monopole antenna before and after the improvement of the meandering technique is simulated and analyzed, and the simulation results are shown in Figure 7. From Figure 7, it can be seen that the VSWR of the antenna in the UHF low band is significantly reduced after the improvement by using the meandering technique compared with that before the improvement. The improved antenna has a VSWR of less than 2 in the band from 570 MHz to 830 MHz, 1.38 GHz to 1.8 GHz and 2.2 GHz to 2.76 GHz, and less than 5 in the band from 450 MHz to 3 GHz, which meets the requirements of GIS PD built-in sensors design.

### 2.3. Flexibility of Monopole Antenna

In order to address the shortcomings of the rigid substrate built-in sensor, this paper introduces the flexible material PI to replace the original rigid substrate material. At present, the main flexible materials studied are polydimethylsiloxane (PDMS) [23], polyetherimide (PI) [24], polyethylene terephthalate (PET) [25], etc. The basic electrical performance parameters of PDMS, PI, and PET flexible materials are shown in Table 2. PI has a low dielectric constant ($\varepsilon_{r}$= 3.5) and dielectric loss (tanδ = 0.008), which can ensure that the antenna has a high signal transmission speed as well as radiation efficiency when used as a dielectric substrate material. At the same time, its excellent electrical insulation, stable chemical properties, and strong mechanical properties make it ideal for use with built-in sensors.

**Figure 8:**
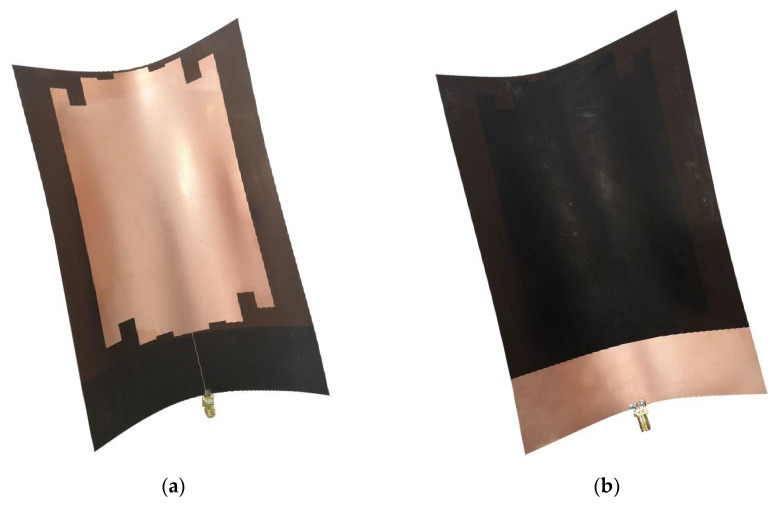
Physical view of the antenna: (**a**) Front side of the antenna; (**b**) Back of the antenna.

In this paper, the monopole antenna optimized by meandering technology was printed on a rectangular PI flexible dielectric board of uniform thickness. The RF connector SMA-KE socket was used to connect the SMA coaxial feed line for feeding. The whole antenna is shown in Figure 8.

## 3. Simulation Analysis of Flexible Antenna Performance

### 3.1. Voltage Standing-Wave Ratio

Voltage Standing Wave Ratio (VSWR) is the ratio of the peak-to-valley value of the traveling standing wave voltage, and the best ratio is 1:1. That is, the input impedance of the antenna is equal to the characteristic impedance of the feed line, which is expressed by the Equation:

(5) $$VSWR=\frac{{\left|U\right|}_{\max}}{{\left|U\right|}_{\min}}=\frac{1+\left|\Gamma \right|}{1-\left|\Gamma \right|}$$

**Figure 9:**
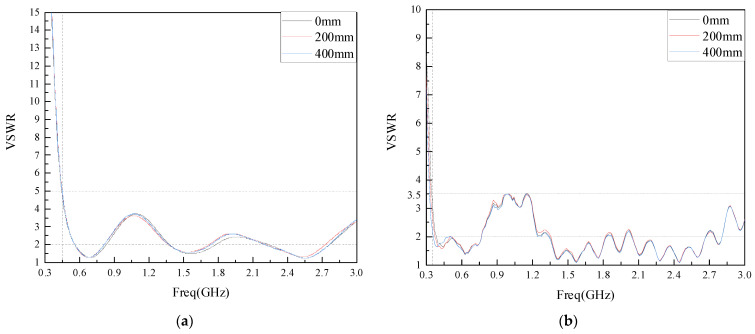
Antenna VSWR under different bending radius: (**a**) Antenna VSWR simulation diagram; (**b**) Antenna VSWR measured diagram.

In Equation (5), Γ is the reflection coefficient at the input of the antenna and represents the ratio of the reflected wave voltage $u_{ro}$ to the incident wave voltage $u_{io}$. When VSWR = 1, i.e., Γ = 0, the antenna reflected wave voltage is 0, and the antenna has no reflection, which is the ideal matching state; when VSWR = ∞, i.e., Γ = 1, the antenna reflected wave voltage is equal to the incident wave voltage, showing total reflection, which is the mismatch state; The internal space of GIS is small, the built-in sensor is close to the discharge source, and the received PD signal is strong. It is generally considered that when VSWR ≤ 5, it is the GIS PD detection bandwidth [26]. As the shell of the actual GIS equipment is mostly a cylinder structure, depending on its voltage level and manufacturing process, the shell bending radius is generally between 150–500 mm. Therefore, this paper simulates the VSWR of the flexible antenna without bending (0 mm) and with bending radii of 200 mm and 400 mm. The simulation results are shown in Figure 9a. As can be seen from Figure 9a, the flexible antenna designed in this paper has a VSWR of ≤5 in the frequency band from 450 MHz to 3 GHz and ≤2 in the frequency band from 570 MHz to 830 MHz, 1.38 GHz to 1.8 GHz and 2.2 GHz to 2.76 GHz when no deformation occurs. The antenna VSWR fluctuates slightly after the bending deformation, but the overall trend remains unchanged.

The VSWR of the physical antenna at different bending radii are tested using a vector network analyzer and the results are shown in Figure 9b. As can be seen from Figure 9b, the VSWR of the antenna designed in this paper is ≤3.5 in the frequency band of 350 MHz–3 GHz. The VSWR changes somewhat after the antenna bends and deforms, but the bending does not change the overall trend of VSWR. Comparing the measured data with the simulation data, it is found that the measured antenna VSWR is better than the simulation data, but the oscillation occurs in the test frequency band, and the overall effect meets the engineering requirements.

### 3.2. Radiation Performance

**Figure 10:**
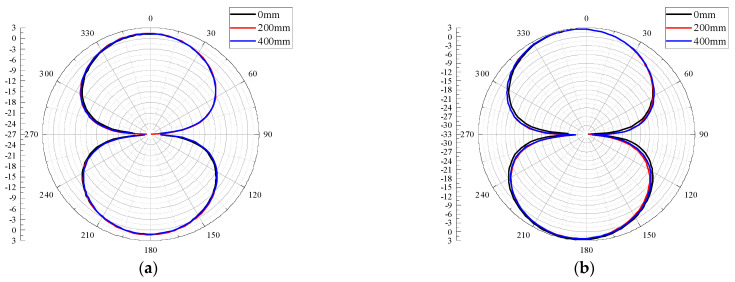

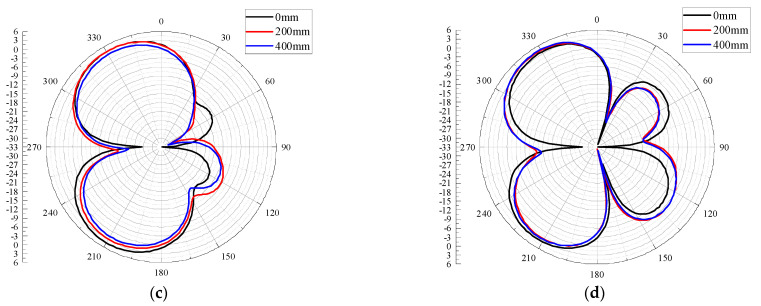
E-plane patterns at different frequencies: (**a**) 0.5 GHz; (**b**) 0.7 GHz; (**c**) 1 GHz; (**d**) 1.5 GHz.

**Figure 11:**
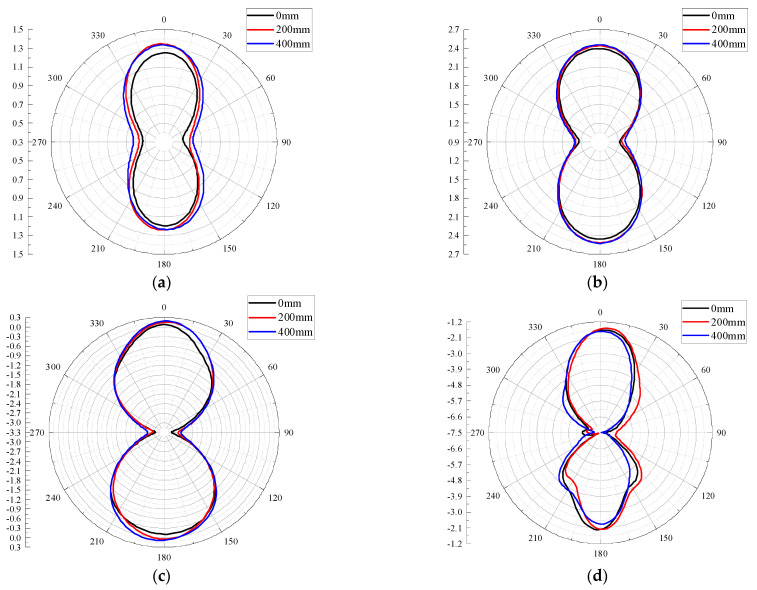
H-plane patterns at different frequencies: (**a**) 0.5 GHz; (**b**) 0.7 GHz; (**c**) 1 GHz; (**d**) 1.5 GHz.

The 2D radiation pattern is a graph showing the relative field intensity and performance variation of the antenna radiation field in different directions within a certain distance from the antenna, usually drawn by polar coordinates. The 2D radiation patterns of the E-planes and H-planes of the antenna designed in this paper are shown in Figure 10 and Figure 11 for four frequency points at 0.5 GHz, 0.7 GHz, 1 GHz, and 1.5 GHz and for different bending radii. It can be seen from Figure 10 and Figure 11 that under different bending radii of the antenna, the E-plane and H-plane patterns at the four frequency points are approximately inverted “8” shapes (a small number of side lobes appear in the high-frequency part), and the antenna can radiate or receive electromagnetic waves in both directions. With the increase in frequency, the antenna E-plane and H-plane patterns have different degrees of degradation; the 1.5 GHz antenna E-plane pattern appears to have side lobes. The reason may be that with the increase in frequency, the higher electromagnetic wave mode will be excited, which distorts the distortion of the patterns.

### 3.3. Flexible Antenna Built-in Simulation Analysis

**Figure 12:**
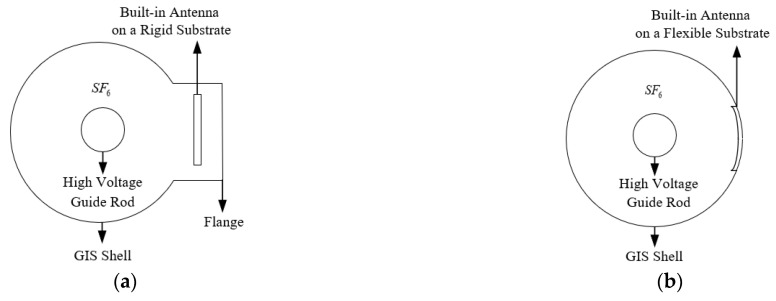
Antenna built-in GIS simplified model diagram: (**a**) Rigid Substrate Built-in Antenna Model; (**b**) Flexible Substrate Built-in Antenna Model.

To analyze the influence of the built-in antennas on the electric field distribution inside GIS with rigid and flexible substrates for GIS PD detection, simplified models of the built-in antennas with rigid and flexible substrates for GIS are built using COMSOL Multiphysics software. For the rigid substrate built-in antenna, the actual installation requires structural modification of the GIS flange, and a simplified model structure diagram is shown in Figure 12a. The real GIS shell (bending radius 325 mm) is set as grounding in the model, and 25 kV is applied to the GIS high voltage guide rod (bending radius 40 mm). The rigid substrate built-in antenna is a simplified model combining an FR4 substrate (thickness 2 mm) and a surface copper layer (thickness 0.1 mm). For the flexible substrate built-in antenna, the actual installation can be conformal with the real GIS inner wall, and the simplified model structure diagram is shown in Figure 12b. The real GIS shell, high voltage guide rod, and applied voltage in the model are consistent with the rigid substrate model. The flexible substrate built-in antenna is a simplified model combining a PI substrate (thickness 2 mm) and a surface copper layer (thickness 0.1 mm).

**Figure 13:**
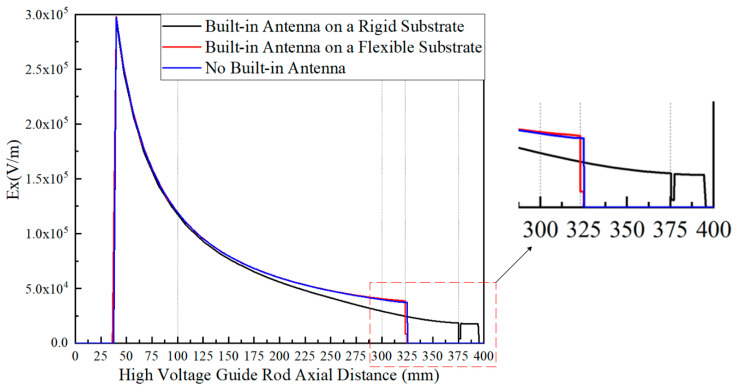
Simulation of the electric field distribution inside the GIS.

The electric field distribution inside the two models of GIS is simulated in the range of 0–400 mm from the axis of the high voltage guide rod, and the electric field distribution inside the GIS without the built-in antenna is simulated in order to analyze the influence of the two material substrates on the electric field distribution, the simulation results are shown in Figure 13.

From the simulation results, it can be seen that for the rigid substrate antenna, in the range of 100 mm–395 mm from the axis of the high voltage guide rod, the internal electric field strength of the GIS of the rigid substrate antenna is less than the electric field strength when the antenna is not placed. The electric field strength in the rigid substrate area (375 mm–377 mm) is less than 5 × 10^3^ V/m. For the flexible substrate antenna, in the range of 0 mm–300 mm from the axis of the high voltage guide rod, the internal electric field distribution of the flexible substrate antenna GIS is the same as the internal electric field distribution when the antenna is not placed, small deviations occur between the two in the 300 mm–323 mm range and the electric field strength at the flexible substrate area (323 mm–325 mm) is less than the electric field strength when the antenna is not placed. In the process of modeling, to better observe the influence of the built-in antenna of the flexible substrate on the electric field distribution inside the GIS, the simplified model parameters of the flexible substrate antenna (PI thickness 2 mm, surface copper layer thickness 0.1 mm) are much larger than the actual parameters of the flexible antenna (PI thickness 0.28 mm, surface copper layer thickness 0.018 mm). Therefore, the influence of the built-in antenna with the flexible substrate on the electric field distribution inside the GIS can be considered negligible. In general, it can be seen from Figure 13 that the built-in flexible substrate antenna keeps the electric field distribution pattern inside the GIS almost unchanged, causing a much smaller impact than that caused by the built-in rigid substrate antenna on the electric field distribution inside the GIS.

## 4. Performance Test Verification of Flexible Antenna

### 4.1. Antenna Bending Deformation PD Detection Performance Test

#### 4.1.1. Construction of Test Platform

**Figure 14:**
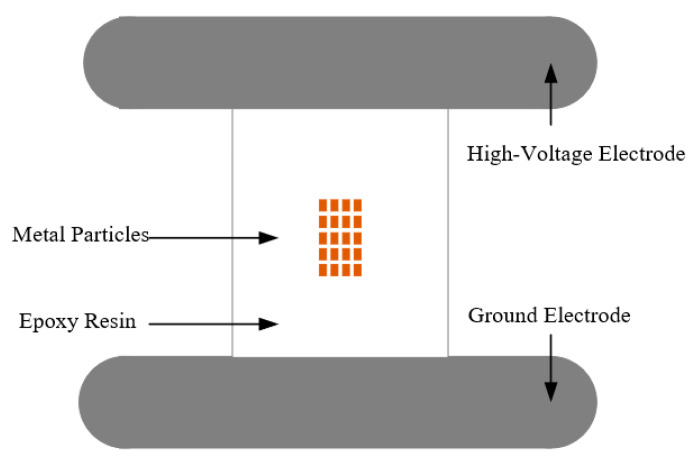
Metal contamination defects model on the insulator surface.

**Figure 15:**
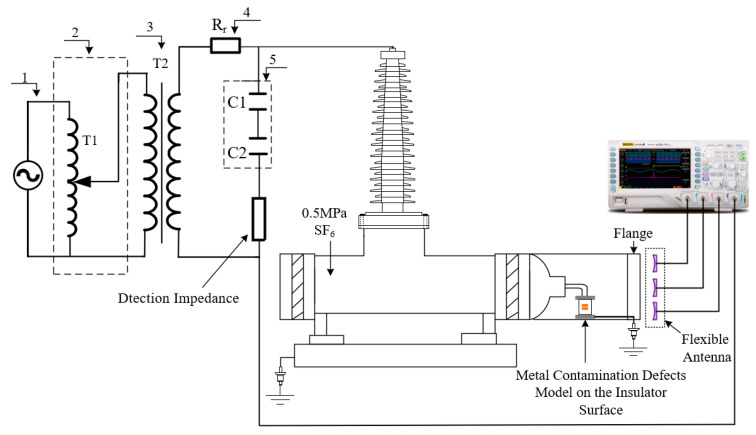
Flexible antenna bending deformation PD detection performance test circuit diagram: 1-High voltage power supply; 2-Voltage regulator; 3-Isolation transformer; 4-Protection resistor; 5-Voltage dividing capacitor.

In order to verify the PD detection performance of the developed flexible antenna after bending and deformation, a power-frequency high-voltage test platform without partial discharge was set up in the laboratory. The actual GIS was filled with 0.5 MPa of SF6 gas, and the metal contamination defects model on the insulator surface was placed inside the GIS to simulate defective discharges. The metal contamination defects model on the insulator surface is shown in Figure 14. The high-voltage electrode was connected to the high-voltage end of the actual GIS through a smooth copper rod, the ground electrode was connected to the ground wire through a metallic copper wire passing through the metallic flange, and metal particles were pasted on the epoxy surface to simulate metal contamination defects on the insulator surface. The metal contamination defects model can simulate the partial discharge phenomenon caused by metal particles on the insulator surface or insulator surface fouling in the actual operation of GIS. Since the bending radius of the curved shell of the laboratory’s actual GIS equipment is fixed, the antenna bending deformation experiment was chosen to be conducted outside the actual GIS equipment. The flexible antennas with a bending radius of 0 mm (no bending), 200 mm and 400 mm, were placed simultaneously at the Plexiglas flange modified by the metal flange. The signal acquisition equipment was a Tektronix* MS044 high-performance digital oscilloscope, which provides 1.5 GHz bandwidth and 6.25 GS/s sampling frequency on all four channels; the test circuit is shown in Figure 15.

#### 4.1.2. Analysis of Test Results

**Figure 16:**
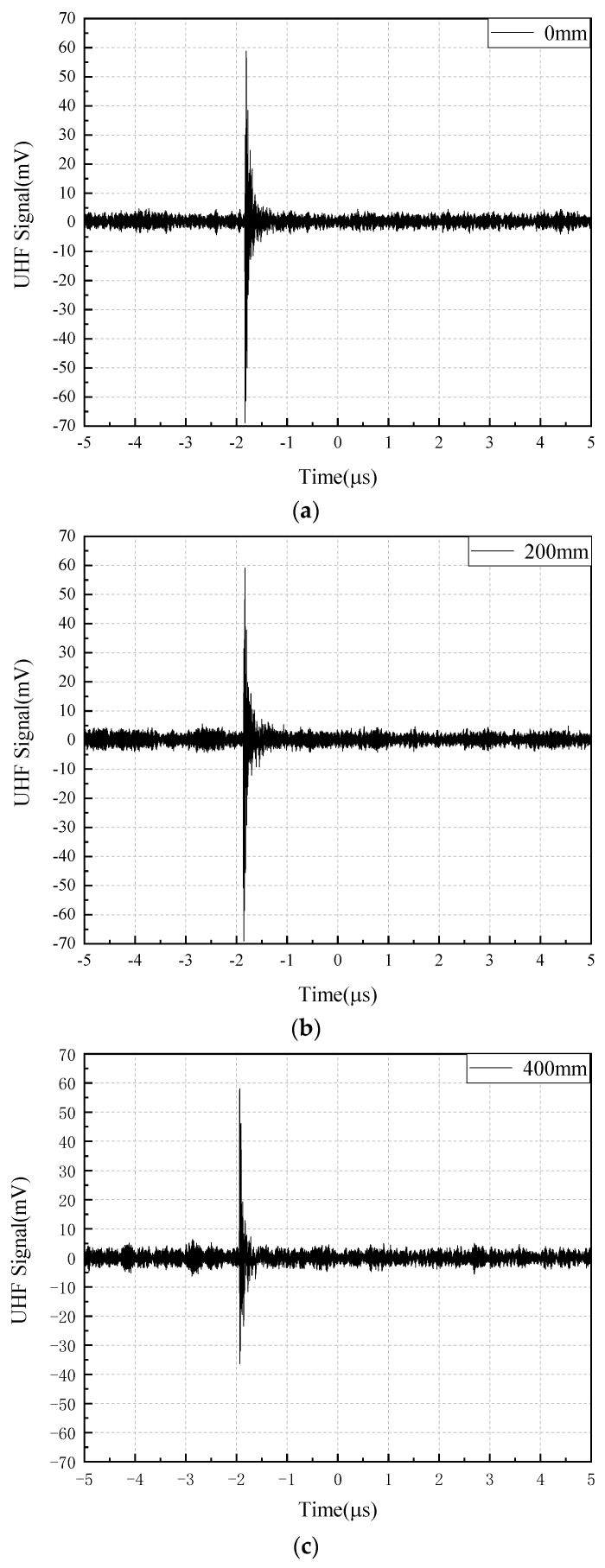
Different bending degree antenna detection PD signal waveform: (**a**) 0 mm; (**b**) 200 mm; (**c**) 400 mm.

Since PD signals were stochastic, 10 PD detection experiments were conducted within the voltage range of 23 kV–26 kV. The amplitude results of PD signals detected by three antennas with different bending radii are shown in Table 3. The average values of PD signal amplitude detected by the three antennas with different bending radii were calculated to be 49.56 mV, 49.85 mV, and 49.59 mV, respectively, and the average values of PD signal amplitude under different bending degrees were approximately the same. The time-domain waveforms of the PD signals detected by the three different bending radii of the flexible antennas are shown in Figure 16 when the experimental voltage is 24 kV and the discharge is 17.4 pC.

**Figure 17:**
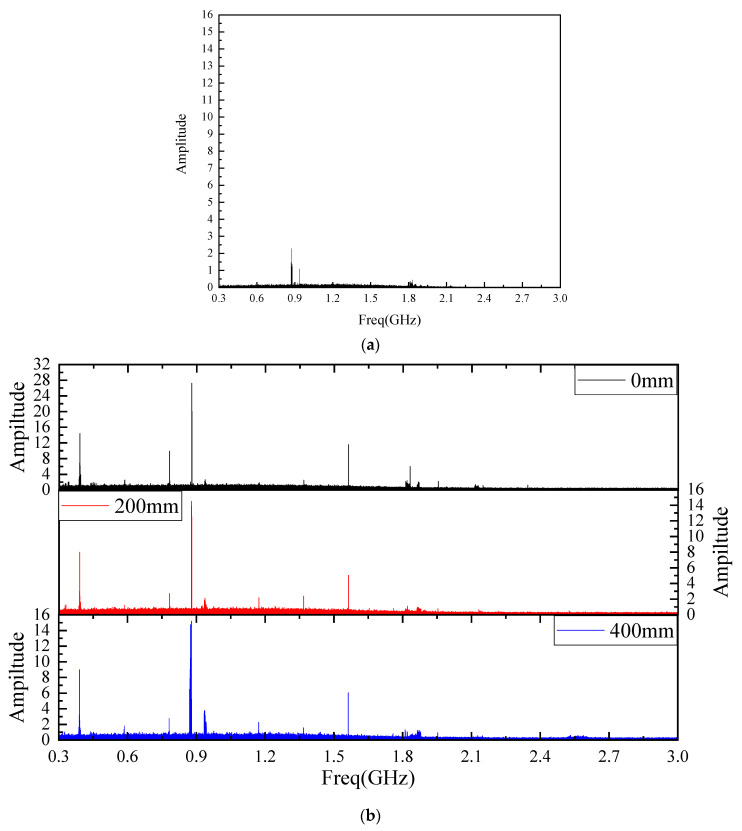
Spectrum Analysis Diagram: (**a**) Electromagnetic Noise Spectrum; (**b**) Spectrum of PD Signals Detected by Antennas with Different Bending Degrees.

It can be seen from Figure 16 that three antennas detect PD signals with different bending degrees, and PD signals are clearly distinguished from background noise. The spectrum of background electromagnetic noise and detected PD signals are analyzed. Figure 17a shows the background electromagnetic noise spectrum, and the PD signals spectrum is shown in Figure 17b. It can be seen from Figure 17a that the frequency points of background electromagnetic noise signals in the experimental environment are concentrated at around 900 MHz and 1.8 GHz, and the frequency points are consistent with 4G communication signals [27]. It can be seen from Figure 17b that PD signals detected by the flexible antenna before and after bending deformation are between 300 MHz and 1.6 GHz, which is consistent with the feature that the main energy of electromagnetic wave signals radiated by GIS discharge defects is distributed between 300 MHz and 1.5 GHz. The amplitude of the PD signals spectrum detected by the antenna after bending is reduced, but the PD signal distribution frequency band is approximately the same. Combined with the time domain waveform and the spectrum analysis, it can be seen that the PD signals can be detected before and after the bending deformation of the flexible antenna, and the PD signals detection performance is the same.

### 4.2. Performance Test of Flexible Antenna Built into the Actual 220 kV GIS

#### 4.2.1. Construction of Test Platform

**Figure 18:**
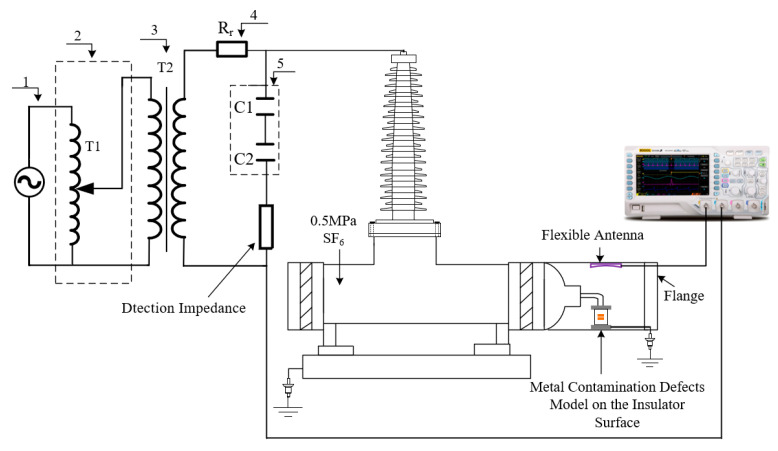
Flexible antenna built into the actual 220 kV GIS test circuit diagram: 1-High voltage power supply; 2-Voltage regulator; 3-Isolation transformer; 4-Protection resistor; 5-Voltage dividing capacitor.

In order to verify the PD detection performance of flexible antenna built into the GIS, this paper carries out the performance test of flexible antenna built into the actual 220 kV GIS. The flexible antenna was fitted to the inner wall of the actual GIS (bending radius 325 mm) and placed. The rest of the test platform is consistent with the flexible antenna bending deformation test, and the test circuit diagram is shown in Figure 18.

#### 4.2.2. Analysis of Test Results

**Figure 19:**
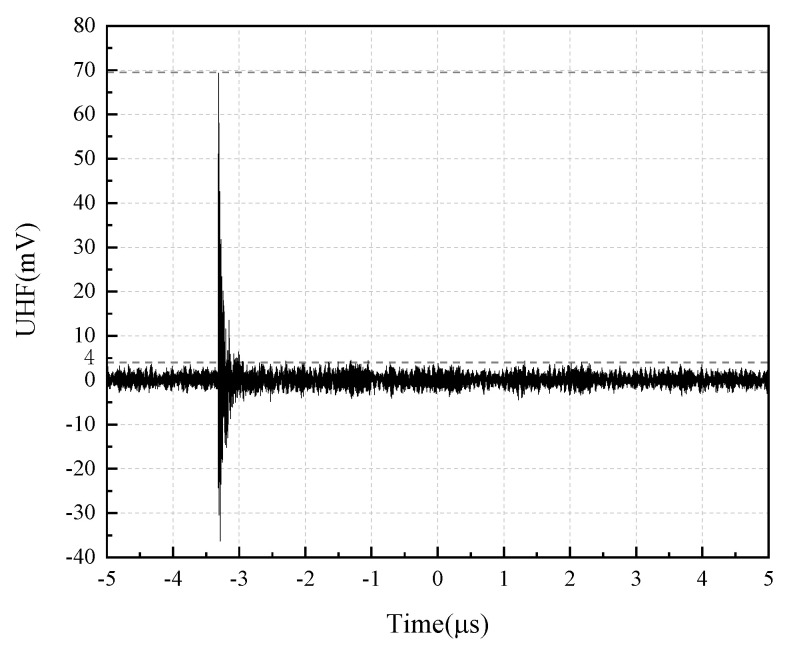
Built-in experimental PD signal waveform.

When the test was pressurized to 23.3 kV, and the discharge was about 13.9 pC, the PD signal waveform was detected, as shown in Figure 19.

**Figure 20:**
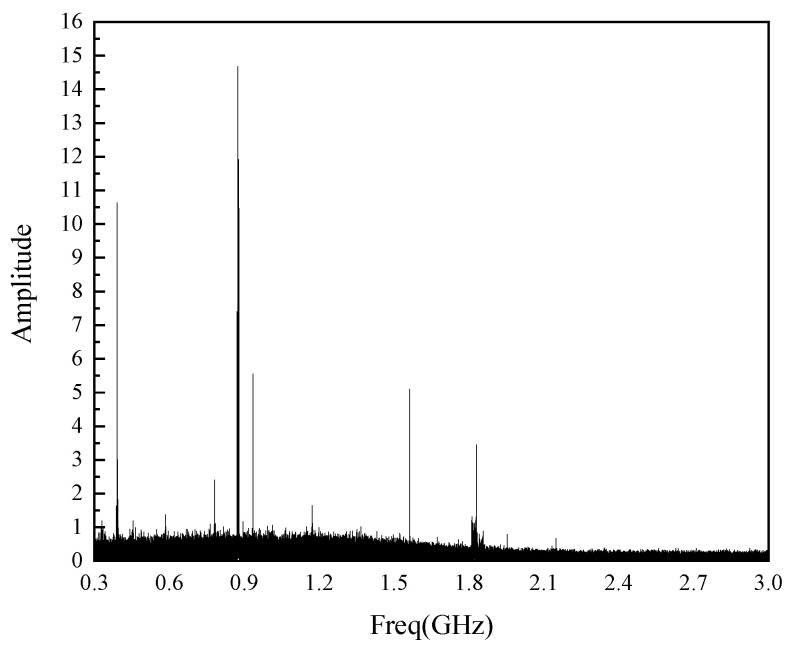
Spectrum diagram of PD signal of built-in experiment.

From Figure 19, it can be seen that the PD signal can be detected after the flexible antenna is built into the true GIS. The detected PD signal amplitude is 69.5 mV, and the background electromagnetic noise signal is below 4 mV, with a high Signal Noise Ratio (SNR). The spectrum analysis of the detected PD signal is carried out, and the result is shown in Figure 20.

From Figure 20, it can be seen that the frequency band of the PD signals detected by the flexible antenna is between 300 MHz and 1.6 GHz, which is the same as that detected by the antennas with different bending degrees in Figure 17b. The built-in experimental PD signal’s time-domain waveform and spectrogram demonstrate the feasibility of PD detection by the built-in GIS of the flexible antenna.

## 5. Conclusions

In view of the low space utilization of the existing built-in antenna sensor for GIS PD detection and the problem of damaging the electric field distribution inside the equipment, this paper carried out the research on the built-in flexible planar monopole antenna for GIS PD detection. PI flexible material is introduced as the antenna substrate, and the performance of the monopole antenna UHF low-frequency band is optimized by using the meandering technique, the PD detection performance of the flexible antenna is verified by experiment, and the following conclusions are drawn:

- In the 300 MHz–3 GHz band, the VSWR of the improved monopole antenna with the curved technology decreases significantly in the UHF low band. Simulation and measurement results show that the designed flexible antenna has a VSWR ≤ 5 in the band of 450 MHz to 3 GHz under different bending degrees, which meets the GIS PD detection requirements of the built-in sensor.
- The COMSOL software is used to simulate the influence of the built-in rigid and flexible substrate antennas on the internal electric field distribution of the GIS. The simulation results show that the built-in flexible substrate antenna keeps the electric field distribution pattern inside the GIS almost unchanged, causing a much smaller impact than that caused by the built-in rigid substrate antenna on the internal electric field distribution of GIS.
- The built PD detection platform is used to conduct bending deformation tests and built-in tests on the flexible antenna. The test results show that the flexible antenna can detect PD signals before and after bending deformation and when it is built into the GIS, proving the feasibility of PD detection on the flexible antenna.

## Figures and Tables

**Table 1:** Optimization results for the five rectangular length parameters.

Slotting Variables	$h_{1}$	$h_{2}$	$h_{3}$	$h_{4}$	$h_{5}$
Paramters (mm)	1.5	4.5	3.4	19.2	10.2

**Table 2:** Basic parameters of flexible materials.

	Material	PDMS	PI	PET
Parameters				
ε_r_		3	3.5	4
tanδ		0.02	0.008	0.04
Breakdown field strength		20 kV/mm	200 kV/mm	380 kV/mm

**Table 3:** PD signal amplitude.

	Experiment Number	1	2	3	4	5	6	7	8	9	10
Bend Radius(mm)		Amplitude (mV)									
0		30.1	39.2	59.8	51.2	41.3	32.8	53.2	69.5	55.4	63.2
200		33.8	38.6	61.5	50.4	42.6	31.5	54.3	68.2	54.8	62.8
400		32.1	38.2	59.2	50.9	43.5	31.9	52.8	68.8	55.1	63.4

## Data Availability

The data presented in the article is original and has not been inappropriately selected, manipulated, enhanced or fabricated by us.
